# Immune recovery uveitis: a focus review

**DOI:** 10.1007/s00417-024-06415-y

**Published:** 2024-02-21

**Authors:** Nuno Rodrigues Alves, Catarina Barão, Catarina Mota, Lívio Costa, Rita Pinto Proença

**Affiliations:** 1 Department of Ophthalmology, Unidade Local de Saúde de São José, Centro Hospitalar Universitário Lisboa Central, Rua José António Serrano, 1150-199 Lisbon, Portugal; 2 NOVA Medical School, Universidade NOVA de Lisboa, Lisbon, Portugal

**Keywords:** Cytomegalovirus retinitis, Human immunodeficiency virus, Immune reconstitution inflammatory syndrome, Immune recovery uveitis, Uveitis

## Abstract

Immune recovery uveitis (IRU) is an intraocular inflammation that typically occurs as part of immune reconstitution inflammatory syndrome (IRIS) in the eye. Typically, it affects human immunodeficiency virus (HIV)-infected patients with recognized or unrecognized cytomegalovirus (CMV) retinitis who are receiving highly active antiretroviral therapy (HAART). IRU is a common cause of new vision loss in these patients, and it manifests with a wide range of symptoms and an increased risk of inflammatory complications, such as macular edema. Recently, similar IRU-like responses have been observed in non-HIV individuals with immune reconstitution following immunosuppression of diverse etiologies, posing challenges in diagnosis and treatment. This review provides an updated overview of the current literature on the epidemiology, pathophysiology, biomarkers, clinical manifestations, diagnosis, differential diagnosis, and treatment strategies for IRU.

## Introduction


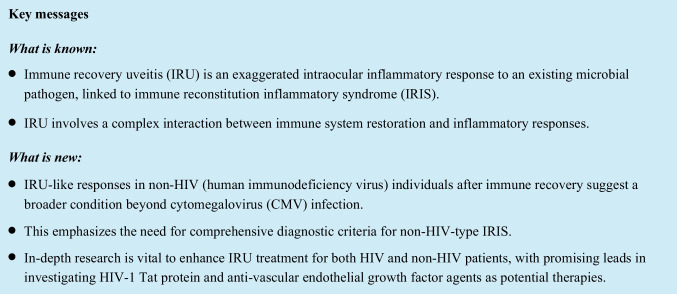


Human immunodeficiency virus (HIV) infection is a retrovirus-induced multisystemic disease that results in the gradual deterioration of the immune system. HIV infection is characterized by the activation of both T and B lymphocytes, leading to the production of inflammatory cytokines. This polyclonal activation of lymphocytes results in the generation of both CD4 + and CD8 + lymphocytes [1]. Inflammatory cytokines such as interleukin-6 (IL-6), interleukin-2 (IL-2), interleukin-1 (IL-1), and tumor necrosis factor (TNF)-alpha further stimulate HIV replication and hasten the progressive destruction of the immune system [1]. As host immune defenses become increasingly compromised, the weakened immune system predisposes infected individuals to opportunistic infections [1, 2].

The introduction of highly active antiretroviral therapy (HAART) marked a pivotal moment in the management of HIV infection, dramatically altering the disease course. This multifaceted approach results in a significant decrease in plasma levels of HIV mRNA and a concomitant increase in CD4 + T-lymphocyte counts, leading to an increased survival rate and a reduction in the incidence of opportunistic infections [1, 3].

In 1998, the American Journal of Ophthalmology published a group of articles that discussed the developing issue of intraocular inflammation among individuals with HIV infection [4]. In patients with CD4 + cell counts lower than 50 cells/μL, it is advised to perform regular ocular evaluations due to the multiple ocular presentations associated with HIV infection [1, 5].

Cytomegalovirus (CMV) is the most common opportunistic infection in late-stage HIV infection [6]. CMV retinitis (CMVR) is associated with a CD4 + T cell count of less than 50/μL and is caused by the dissemination of the virus through retinal blood vessels following hematogenous spread after the reactivation of latent CMV infection [6, 7]. In patients with HIV and CMVR, ocular inflammation is typically minimal or absent, which can be attributed to the underlying immunosuppressed state [8]. The diagnosis of CMVR is usually straightforward, as it presents as centripetal necrotic retinal areas with associated hemorrhage, known as “pizza retinopathy,” variable small dot-like lesions, and retinal vasculitis with perivascular sheathing [9]. CMVR causes full-thickness retinal necrosis, which leaves an atrophic scar and may result in decreased visual acuity [7, 10].

Notably, the incidence of CMVR has decreased by as much as 90% with the advent of HAART therapy [7, 11]. A response to HAART, also known as immune recovery, is defined as an increase in CD4 + T cell count of at least 50 cells/μL, with a target level of 100 cells/μL or more [12].

Although it has reduced the prevalence of opportunistic infections, HAART has also increased the incidence of immune reconstitution inflammatory syndrome (IRIS) [13, 14]. This phenomenon is characterized by the paradoxical worsening of a treated opportunistic infection or the unmasking of a previously subclinical, untreated infection in patients with HIV following the initiation of HAART due to a tissue-destructive inflammatory response [15]. The incidence of IRIS in patients with acquired immunodeficiency syndrome (AIDS) receiving HAART ranges from 10 to 32% [13, 14].

The current definition of IRIS includes five criteria: (1) confirmation of HIV infection, (2) temporal association between IRIS development and HAART initiation, (3) specific host responses to HAART, such as a decrease in plasma levels of HIV RNA and an increase in CD4 + cell count, (4) clinical deterioration characterized by an inflammatory process, and (5) exclusion of other causes. Pathogens associated with IRIS include *Mycobacterium tuberculosis,* atypical mycobacterium*,* CMV, Varicella-zoster virus*, Pneumocystis jirovecii, Toxoplasmosis gondii,* and *Cryptococcus neoformans* [1, 16].

The majority of cases of IRIS occur during the first three months of HAART, although it can appear later, generally 3–12 months after HAART initiation [17]. A few cases occurring up to four years later have been described [18].

Ocular IRIS is referred to as immune recovery uveitis (IRU), which remains a leading cause of ocular morbidity [1]. IRU is the most common form of IRIS in HIV-infected patients with CMVR who are receiving HAART [1, 19]. The underlying pathogenesis of IRU is not yet fully comprehended. It is generally accepted that IRU is a response against CMV within the eye, and it is a paradoxical intraocular inflammation that occurs following CMVR [20, 21]. This immune recovery is associated with a heightened incidence of inflammatory complications, including macular edema [1].

In general, HAART is recommended to be started immediately after the diagnosis of HIV. However, a 2005 study suggested that delaying HAART may result in a reduction in the frequency and severity of CMV-associated IRU [22].

Although IRU can manifest in patients long after the initiation of HAART, it is hypothesized that incomplete CMVR resolution with subclinical production of viral proteins can persistently stimulate the immune system in some patients, leading to the development of IRU [23]. Thus, maintenance of anti-CMV therapy following the initiation of HAART may reduce the incidence and severity of IRU. However, despite anti-CMV therapy, IRU may still occur in these patients [23].

## Epidemiology

IRU has been recognized as a significant contributor to vision loss in patients with CMVR related to HIV [24]. The incidence of IRU varies among studies, and this variability may be attributed to various factors, including the era of HAART therapy [13, 22, 25–27]. Studies have shown that IRU affects 10% of patients with CMVR in the early HAART era [25]. In a retrospective study, IRU was observed in 17.4% of patients receiving HAART. Of these patients, more than half developed IRU with only a minimal increase in CD4 counts of approximately 100 to 150 cells/mm3 [13]. A prospective multicenter observational study conducted in the modern HAART era revealed that the incidence of IRU was 1.7 to 2.2 per 100 person-years (PY) and was correlated with higher rates of vision loss and blindness when compared to individuals who had immune recovery without IRU [28].

Several factors may contribute to the lower incidence of IRU in some studies, including the use of more aggressive anti-CMV therapy before and after initiating potent antiretroviral therapy, which may reduce exposure to CMV antigens during the critical period of immune recovery in the eye [23]. Another contributing factor could be the use of intravitreal cidofovir, a significant risk factor for IRU that was utilized in the treatment of CMVR in older studies [26].

The decision-making process regarding the timing of HAART in the presence of CMVR is complex. Early introduction of HAART before completing induction therapy for CMV may result in a higher incidence of IRU [1, 7]. Controlling CMVR before starting HAART can significantly reduce the occurrence and severity of IRU [22]. Therefore, continuing anti-CMV treatment to minimize lesions until the immune system is strong enough to control retinitis is necessary [23]. However, it is essential to recognize the nuanced nature of this decision. IRIS develops as the immune system recovers from a low CD4 count. This underscores the importance of considering the initiation of HAART before CMVR develops to prevent a low CD4 count [1, 3].

Despite the availability of HAART, patients with HIV and CMVR remain at an increased risk for mortality, retinitis progression, complications of retinitis, and visual loss over five years [1].

## Pathophysiology and biomarkers

The eye is considered an immunological sanctuary due to the blood-aqueous and blood‒retina barriers, composed of retinal microvascular endothelial cells and retinal pigment epithelium (RPE), which prevent large molecules, cells, and microorganisms from entering the eye [21]. In individuals with HIV, ocular complications can occur due to the breakdown of the blood‒retinal barrier (BRB), facilitating the leakage of CMV antigens within the eye. This phenomenon may or may not be associated with viremia status in HIV patients, enabling CMV antigens to access lymphoid organs and trigger an antigen-specific immune response [1, 6]. The underlying mechanism for this breakdown is not fully understood, but exposure of retinal neurosensory and glial cells to HIV Tat, the transactivator protein of HIV-1, has been shown to result in increased activation and release of proinflammatory mediators [29]. Additionally, HIV-1 Tat protein has been found to induce apoptosis in human retinal microvascular endothelial cells and RPE cells [29].

As immune function improves with HAART, a threshold is reached at which the body can mount an intraocular inflammatory response to CMV antigens present in the eye. As immune function continues to recover, the threshold is elevated, leading to the inactivation of CMV by the immune system. This results in the cessation of CMV replication, reducing the antigen load, and subsequently, the inflammatory reactions subside [4].

Several biomarkers have been identified over the years. Immunohistological examination of epiretinal membrane (ERM) associated with IRU has shown evidence of chronic inflammation with a predominant T-lymphocyte presence, suggesting that IRU may be due to a T cell-mediated reaction to CMV antigen [30, 31]. Samples of aqueous and vitreous fluids from patients with IRU and CMVR were examined for the presence of cytokines and CMV DNA. Inflammatory IRU can be differentiated from active CMVR by the presence of IL-12 and less IL-6 and the absence of detectable CMV replication [6, 32].

Furthermore, a unique immunologic signature of cytokine-mediated inflammatory response pattern (IP-10, PDGF-AA, G-CSF, MCP-1, fractalkine, Flt-3L) is actively present in the aqueous humor [33]. Reports have suggested that all patients with clinical and ophthalmological characteristics of IRU show the presence of HLA-A2, HLA-B44, HLA-DR4, and HLA-B8-18 [12, 34]. The expression of miRNA-192 in patients with IRU was significantly decreased, which may help identify the status of people living with HIV [35].

A multicenter observational study found that patients with IRU had weaker antiviral CD4 + T cell responses than control subjects who did not develop IRU, while CD8 + T cell responses were comparable. Patients with IRU also had a smaller number of Th17 cells, which may reflect greater losses throughout the course of HIV disease and a greater level of immune dysfunction. In their opinion, CD4 cell count and Th17 cell number may both be measures of the severity of HIV disease before the initiation of HAART [36]. Three metabolites were identified as the most specific indices to distinguish IRIS before HAART: (i) oxidized cysteinyl-glycine (Cys-Gly Oxidized), (ii) 1-myristoyl-2-palmitoyl GPC (14:0/16:0), and (iii) the sulfate of piperine metabolite (C18H21NO3) [37]. However, after 1 month of HAART, quinolinate, gluconate, and serine were the indices driving the distinction [37]. These serum metabolites are specific markers for systemic IRIS and are not explicitly mentioned for uveitis [37]. However, as IRU is part of IRIS, these metabolites may also serve as biomarkers for IRU, although no previous studies have described specific metabolites for IRU. Additionally, plasma TNF-α and mucin domain 3 levels were significantly increased after HAART in HIV-infected patients with IRIS [38].

## Clinical presentation

IRU is a clinical entity with variable manifestations, ranging from anterior uveitis to severe vitritis, with potential complications that can significantly impact visual function. The severity of inflammation is influenced by several factors, including the degree of immune reconstitution, the extent of CMVR, the amount of intraocular CMV antigen, and previous treatment [1].

**Fig. 1:**
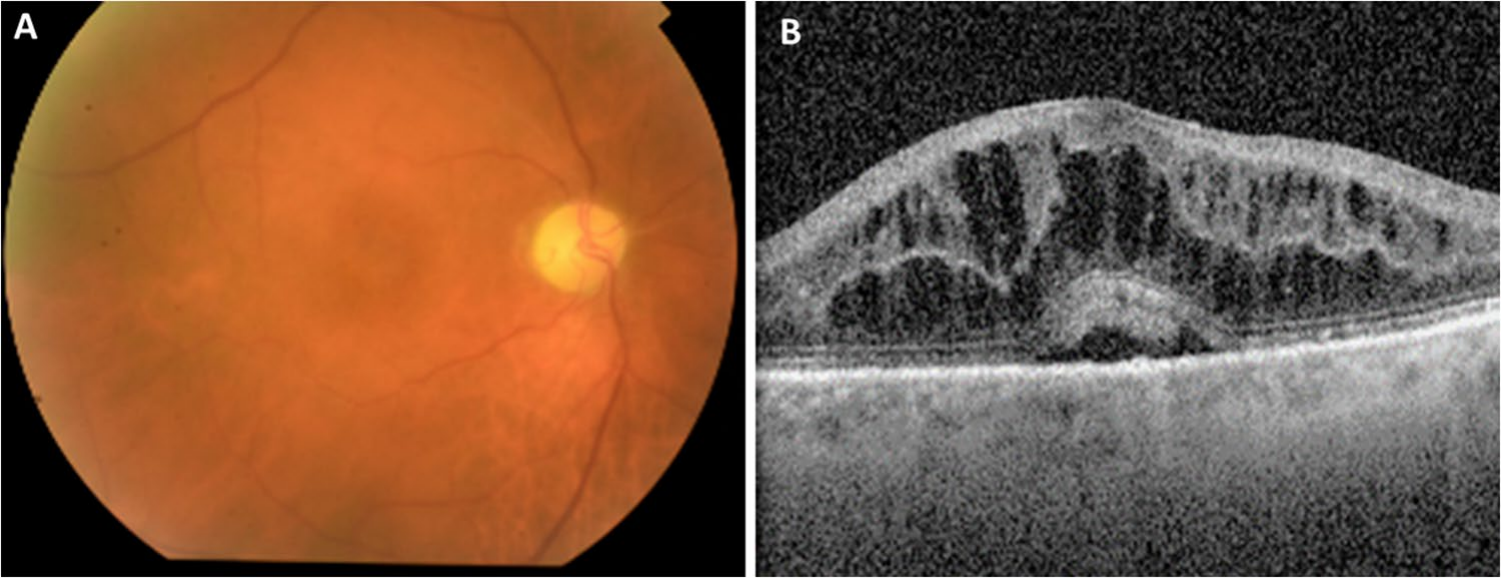
Clinical presentation of IRU. **A** A fundoscopy with discrete vitritis, macular edema, optic disc pallor and peripheral atrophic chorioretinal scars with no signs of activity of CMVR in one 71-year-old patient with microscopic polyangiitis treated with mycophenolate mofetil referred for observation due to complaints of left reduced visual acuity and floaters that started ten months after suspension of immunosuppressive therapy due to CMV colitis. **B** The optical coherence tomography (OCT) of the same patient with cystoid macular edema

Typical symptoms of IRU include floaters and moderate visual acuity loss (usually worse than 20/40 but better than 20/200) [1]. Following initiation of HAART and increasing CD4 + lymphocyte counts, an anterior chamber inflammatory reaction and vitreous haze (Fig. 1A) may develop within weeks [5, 19]. However, this stage can be transient and easily missed [8].

Mostly, patients with IRU do not have active CMVR, as the improved immune function that leads to inflammation also enables better control of the CMVR. In some cases, the infection may remain clinically inactive even without specific anti-CMV therapy [39].

Complications associated with IRU include cystoid macular edema (CME) (Fig. 1B), which is the leading cause of vision loss in patients with IRU [1]. A study found that patients with IRU had a 20-fold higher risk of CME than those without IRU [25]. Other potential clinical manifestations or complications of IRU include anterior segment inflammation, synechiaes, cataracts, vitreomacular traction, ERM formation, macular hole, proliferative vitreoretinopathy with risk of retinal detachment, frosted branch angiitis, papillitis, neovascularization of the retina or optic disc, severe postoperative inflammation, and uveitic glaucoma [1, 5, 21, 40].

The risk of IRU-induced complications is proportional to the absolute difference in CD4 + counts between the start of HAART and the development of IRU [41]. Therefore, close monitoring of CD4 + counts and regular ophthalmologic evaluations are essential for early detection and management of IRU and its associated complications.

## Diagnosis and differential diagnosis

IRU is an exclusion diagnosis that is typically associated with HIV patients receiving HAART who have experienced an increase in their CD4 + T cell count > 100 cells/mm3 and have a new paradoxical inflammation in an eye with a preexisting history of CMVR or other ocular infection. The current definition of IRU requires the presence of intraocular inflammation that cannot be explained by drug toxicity or a new infection, for example, cidofovir toxicity, rifabutin toxicity, syphilitic uveitis, ocular tuberculosis, toxoplasmosis retinochoroiditis, and the development of new sarcoidosis [1, 9]. In certain immunocompromised patients, as the immune system undergoes recovery, an immune response may occur, giving rise to an IRU-like syndrome. Diagnostic testing, such as multimodal imaging, ocular polymerase chain reaction (PCR), and diagnostic vitrectomy, is essential in cases where the differential diagnosis includes inflammatory, infectious, and neoplastic processes [42]. Furthermore, IRU-like responses have been observed in HIV-negative individuals with recovery from immunosuppression due to other etiologies, including lymphoma, Wegener granulomatosis, Good Syndrome, rheumatoid arthritis, leukemia, and after renal and bone marrow transplant [42–53]. Mostly, immunosuppressive treatment reduction is associated with the manifestation of IRU-like syndromes. Table 1 provides a comprehensive overview of clinical cases documenting IRU in non-HIV patients from the literature.

**Table 1:** IRU in non-HIV case reports

Source	Predisposing condition	Pre-existing history of ocular infection	Occurrence of IRU
Kuo, 2004 [53]	Bone marrow transplant	CMV	After lowering the imunnosupressive therapy
Kuo, 2004 [53]	Interstitial lung disease	CMV	After lowering the imunnosupressive therapy (cyclophosphamide)
Kuo, 2004 [53]	Cardiac transplant	CMV	After lowering the imunnosupressive therapy
Kuo, 2004[53]	Systemic necrotizing vasculitis	CMV	After lowering the imunnosupressive therapy (cyclophosphamide)
Miserocchi, 2005 [47]	Renal transplant	CMV	After lowering the imunnosupressive therapy (cyclosporin and prednisone)
Baker, 2006 [45]	Non-Hodgkin's lymphoma	CMV	After tapering immunosuppression (cyclosporine and mycophenolate) to prevent graft rejection of bone marrow transplant
Bessho, 2007 [46]	Wegener's granulomatosis	CMV	After lowering the immunosuppressive therapy (cyclophosphamide and prednisone)
Wimmersberger, 2011 [51]	Chronic lymphatic leukaemia	CMV	After chemotherapy cessation
Agarwal, 2014 [52]	Acute lymphoblastic leukaemia	CMV	After lowering the imunnosupressive therapy
Agarwal, 2014 [52]	Acute lymphoblastic leukaemia	CMV	After lowering the imunnosupressive therapy
Agarwal, 2014 [52]	Acute lymphoblastic leukaemia	CMV	After lowering the imunnosupressive therapy
Agarwal, 2014 [52]	Renal transplant	CMV	After lowering the imunnosupressive therapy
Downes, 2016 [50]	Good syndrome	CMV	During anti-CMV therapy
Yanagisawa, 2017 [49]	Rheumatoid arthritis	CMV	MTX + TOF regimen was stopped due to CMVR. IRU surged during anti-CMV treatment
Lavine, 2018 [42]	Angioimmunoblastic T-cell lymphoma	Non-detectable	After chemotherapy and autologous bone marrow transplant therapy
Sánchez-Vicente, 2019 [44]	Hairy cell leukaemia	HSV	After the last cycle of pentostatin
Tai, 2021 [48]	Good syndrome	CMV	After multiple doses of GCSF
Saricay, 2023 [43]	Acute leukaemia	CMV	During concurrent chemotherapy and systemic antiviral therapy

*CMV* cytomegalovirus, *CMVR* cytomegalovirus retinitis, *GCSF* granulocyte colony-stimulating factor, *HIV* human immunodeficiency virus, *IRU* immune recovery uveitis, *MTX* methotrexate, *TOF* tofacitinib

In summary, IRU is a clinical diagnosis that is based on a set of exclusion criteria and a combination of clinical and laboratory findings and requires careful assessment by a multidisciplinary team.

## Risk factors

Several risk factors are associated with the development of IRU:

- Immune recovery with a rapid rise in CD4 + T cell count (specifically, a CD4 + count 100 to 199 cells/microliter) (Odds ratio (OR 21.8) [25];
- Intravitreal cidofovir (OR 19.1) [25]
- CMVR extension area of ≥ 25% (OR 2.52) [25].

On the other hand, the presence of a posterior pole lesion (area including a 1500-µm radius around the optic nerve and a 3000-µm radius around the center of the macula; OR 0.43), male gender (OR 0.23), and HIV load > 400 copies/milliliter (OR 0.26) were found to be associated with reduced IRU risk [25].

Other factors that are currently unidentified might impact the susceptibility and severity of IRU. Therefore, additional research is warranted to identify and understand these factors better.

## Treatment

The management of IRU involves considering various factors, including the extent and activity of the underlying CMV retinitis, the presence or absence of non-ocular IRIS, the site of intraocular inflammation, the existence of any associated ocular complications, particularly macular edema, and the patient’s general health [1].

### Medical treatment

Topical corticosteroids are typically used for anterior chamber inflammation, while mild vitritis without macular edema may simply be observed [54, 55].

Short courses of oral corticosteroids or sub-Tenon steroid injections or periocular corticosteroids (e.g., triamcinolone acetonide) are reserved for cases of severe vitritis and/or CME, but their effectiveness is limited [31, 54]. Fluocinolone acetonide intravitreal implants have shown promising improvements in CME associated with IRU [56]. Intravitreal corticosteroids (e.g., dexamethasone) may be considered in refractory cases, but caution must be taken due to the risk of increased intraocular pressure, cataracts, and CMVR reactivation, which can be prevented by restarting anti-CMV therapy [19, 54, 57]. Additionally, steroid use is a significant risk factor for the occurrence of tubercular uveitis, so it is important to monitor for this condition [58, 59].

Discontinuing antiretroviral therapy is not recommended because it reduces the CD4 + T lymphocyte count and increases the risk of opportunistic infections [21]. Patients with CMVR who are receiving antiretroviral regimens should continue with anti-CMV maintenance therapy (Table 2) until the IRU is resolved and sustained immune recovery is achieved (CD4 + T lymphocytes ≥ 100 cells/µL for 3–6 months) [21]. Even after discontinuation of anti-CMV therapy, patients with a history of CMVR should be closely monitored at 3-month intervals, as they remain at risk for recurrence [21]. International guidelines recommend initiation of HAART within two weeks of commencing anti-CMV therapy in HIV/CMVR patients, although these recommendations are based on expert opinions and not empirical evidence [60, 61].

**Table 2:** Treatment recommendations for CMVR. Adapted from Port et al., (2017) [9]

Recommended treatment regime	Alternate regime	Remarks
*Induction* -Oral Valganciclovir 900 mg twice daily -Intravenous Ganciclovir 5 mg/kg twice daily for 14–21 days -Intravitreal Ganciclovir 2 mg/0.1 mL twice weekly *Maintenance* -Oral Valganciclovir 900 mg daily -Intravenous Ganciclovir 5 mg/kg/day -Intravitreal Ganciclovir 2 mg/0.1 mL weekly	*Induction* - Intravenous Foscarnet 90 mg/kg twice daily for 14 days -Intravenous Cidofovir 5 mg/kg weekly for 3 weeks *Maintenance* -Intravenous Foscarnet 120 mg/kg/day -Intravenous Cidofovir 5 mg/kg every 2 weeks *Intravitreal Induction* -Foscarnet 1.2–2.4 mg 1–2 times weekly -Cidofovir 20 μg 1–8 times as needed to halt retinitis *Intravitreal Maintenance* -Foscarnet 1.2 mg weekly -Cidofovir 20 μg every 5–6 weeks	Ganciclovir/Valganciclovir: Myelosuppression Foscarnet: Nausea and vomiting, electrolyte disturbance, nephrotoxicity Cidofovir: nephrotoxicity, anterior uveitis CMV mutations in UL54 or UL97 genes cause ganciclovir, foscarnet and cidofovir resistance [62]

Of note, Cidofovir should not be used if immune recovery is expected due to its association with IRU

Furthermore, the discoveries regarding the involvement of the HIV-1 Tat protein in disrupting the integrity of the BRB suggest that blocking the activity of HIV-1 Tat could be a crucial component of future therapeutic approaches for managing CME associated with IRU [63].

More recently, some studies have demonstrated the effectiveness of anti-vascular endothelial growth factor (VEGF) agents in treating IRU-induced CME, especially aflibercept [64]. Increased levels of proangiogenic factors such as VEGF and placental growth factor (PlGF) have been reported in ocular inflammation, and these factors can promote cell proliferation, migration, and vascular permeability [65]. Intraocular bevacizumab and aflibercept have anti-inflammatory effects, and aflibercept has a higher affinity for VEGF-A, VEGF-B, and PlGF than bevacizumab and ranibizumab [66, 67]. These differences may be responsible for the better effect of aflibercept in treating IRU-induced CME [64].

A substantial challenge arises in balancing treatment decisions, notably when facing complications like macular edema refractory to diverse interventions, even amid sustained immune recovery. This intricate balancing act necessitates a nuanced consideration of risks and benefits associated with off-label treatments.

### Surgical management

Surgical treatment plays an important role in managing various retinal conditions and can significantly improve visual outcomes for patients. Vitreomacular traction syndrome, ERM, cataract, and proliferative vitreoretinopathy are some of the possible indications for surgery [1].

## Discussion

This comprehensive and updated review provides an in-depth analysis of IRU, covering various aspects, including epidemiology, pathophysiology, biomarkers, clinical presentation, risk factors, diagnosis, differential diagnosis, and treatment. The findings offer valuable insights for clinical practice and highlight potential areas for future research in the field of IRU.

Immunosuppression, especially when HIV patients have a CD4 + cell count lower than 50 cells/μL, is strongly associated with an increased risk of diverse ocular presentations [12]. This underscores the critical importance of regular ocular evaluations and screening for visual impairment in this population [1, 5]. Among the opportunistic infections observed in late-stage HIV infection, CMVR emerges as the most common ocular manifestation [6, 7]. While HAART has proven effective in reducing opportunistic infections, it has also been associated with an increased incidence of IRIS [11, 13, 14].

IRIS is a tissue-destructive inflammatory response characterized by the improvement of a previously incompetent human immune system, resulting in the worsening of clinical symptoms due to a vigorous inflammatory response [15, 68]. Initially, recognized in the context of HIV infection, IRIS continues to be most frequently encountered in this clinical setting [69]. This highlights the complex interplay between immune reconstitution and inflammatory responses in HIV patients.

Notably, despite the implementation of maintenance anti-CMV therapy following HAART initiation, cases of IRU can still occur, suggesting that the underlying mechanisms driving IRU might extend beyond CMV infection alone [23]. Interestingly, some patients develop IRU with only a minimal increase in CD4 + counts, indicating that IRU can manifest even in individuals with modest immune recovery. This underscores the need for vigilant monitoring and early detection of IRU in HIV-infected patients, irrespective of the magnitude of CD4 + cell count improvement.

Optimal timing is crucial in the management of IRU. Initiating HAART before completing induction therapy for CMVR may increase the risk of developing IRU [1, 22]. Expert opinions recommend initiating HAART within two weeks of commencing anti-CMV therapy in HIV/CMVR patients [60, 61]. Furthermore, the risk of IRU-induced complications appears to correlate with the absolute difference in CD4 + counts between HAART initiation and IRU development, emphasizing the significance of regular monitoring and ophthalmologic evaluations to promptly identify potential complications [41].

An accurate diagnosis of IRU relies on a set of exclusion criteria and a comprehensive assessment incorporating clinical and laboratory findings [1, 9]. It is crucial to differentiate IRU from other ocular conditions, as this has significant implications for appropriate management. Complex cases may require advanced diagnostic modalities such as multimodal imaging, ocular PCR, and diagnostic vitrectomy to aid in the differential diagnosis [42].

While IRU has primarily been studied in HIV-infected patients, it is noteworthy that similar IRU-like responses have been observed in patients with diverse etiologies when immunosuppression is reversed [70]. This broader spectrum of conditions associated with IRU-like responses, ranging from lymphoma to hematopoietic stem cell transplantation, highlights the multifactorial nature of this inflammatory phenomenon [42–53]. This suggests that IRU may be a broader spectrum condition than currently described, as several cases have occurred in non-HIV patients with different previous ocular infections, challenging our understanding of IRU. In addition to CMV, ocular IRIS can be observed with other ocular opportunistic infections, including ocular tuberculosis and *Cryptococcus neoformans* [71, 72].

The establishment of the concept, definition, and diagnostic criteria of non-HIV-type IRIS would aid clinical decision-making regarding the continuation of treatment for the original disease. This new concept has the potential to benefit patients by providing tailored management strategies. Further complexity emerges in the absence of fluoxograms, challenging practitioners in their diagnostic and management toolkits.

Treatment strategies for IRU primarily aim to control inflammation and preserve visual function, with steroids being the standard therapy [54, 55]. Ongoing anti-CMV therapy can be beneficial in preventing CMVR reactivation, and diligent monitoring is essential for patients with a history of CMVR [7, 54, 57]. Antiretroviral therapy should not be discontinued, as it increases the risk of opportunistic infections and compromises immune recovery [21]. Patients with CMVR who are receiving antiretroviral regimens should continue with anti-CMV maintenance therapy until IRU has resolved and sustained immune recovery is achieved, described as CD4 + T lymphocytes ≥ 100 cells/μL for 3–6 months [21].

Exciting research has highlighted the involvement of the HIV-1 Tat protein in disrupting the BRB, suggesting that targeting this protein may hold promise for managing CME associated with IRU [63]. Moreover, studies have demonstrated the effectiveness of anti-VEGF agents, particularly aflibercept, in treating IRU-induced CME [64–67]. These emerging therapeutic avenues offer potential breakthroughs in the management of IRU and warrant further investigation. However, considering aspects related to cytokine biomarkers and the inflammatory pathophysiology of IRIS, the use of TNF inhibitors, anti-IL-6, or other biological treatments for IRU remains questionable [73]. Controlled studies supporting the use of these pharmacologic interventions in IRIS and IRU are limited, and current recommendations are largely based on case series.

## Conclusion

In summary, IRU is an intraocular inflammation and may be the sole manifestation of IRIS, involving a complex interplay between immune reconstitution and inflammatory responses. Notably, IRU can occur even in individuals with modest immune recovery and may have underlying mechanisms beyond CMV infection alone. Therefore, diligent monitoring and early detection of IRU are essential, considering the potential for diverse etiologies of immunosuppression.

Accurate diagnosis of IRU relies on comprehensive assessments that incorporate clinical and laboratory findings, possibly utilizing advanced diagnostic modalities when necessary. The broad spectrum of conditions associated with IRU-like responses challenges our current understanding and emphasizes the need for a comprehensive definition and diagnostic criteria for non-HIV-type IRIS. Such an approach would facilitate clinical decision-making and enable tailored management strategies for patients.

Further research is warranted to optimize treatment approaches, explore novel therapeutic targets, and improve patient outcomes for both HIV and non-HIV individuals affected by IRU. Advancements in these areas will contribute to better understanding, management, and ultimately, the overall well-being of patients affected by this complex inflammatory condition.
